# Evanescent Wave-Based Photonic Biosensors for the Design and Evaluation of Advanced Cancer Immunotherapies

**DOI:** 10.1021/acs.analchem.5c07797

**Published:** 2026-02-27

**Authors:** César S. Huertas, Laura M. Lechuga, Maria Soler

**Affiliations:** † Integrated Photonics and Applications Centre (InPAC), School of Engineering, RMIT University, Melbourne 3001, Australia; ‡ Nanobiosensors and Bioanalytical Applications Group (NanoB2A), Catalan Institute of Nanoscience and Nanotechnology (ICN2), CSIC, CIBER-BBN and BIST, 08193 Bellaterra (Barcelona), Spain

## Abstract

Personalized immunotherapies hold great promise for correcting cellular dysfunction, inhibiting tumor growth, and even achieving durable cancer eradication. However, conventional analytical methods used to design and evaluate immunotherapies often fall short as they typically cannot monitor cell activity in real time, lack multiplexed capabilities for rapid screening, and require complex sample preparation. These limitations impede a full understanding of the dynamic immune responses that drive therapeutic outcomes. Label-free optical biosensors based on evanescent wave interactions provide a compelling alternative, enabling sensitive, noninvasive, and high-resolution analysis of cellular regulation, signaling, and therapy-induced molecular changes. In this perspective, we highlight recent advances in optical biosensor technologies for molecular and cell analysis and explore their potential to accelerate the development, optimization, and precision application of next-generation immunotherapies.

## Graphical abstract

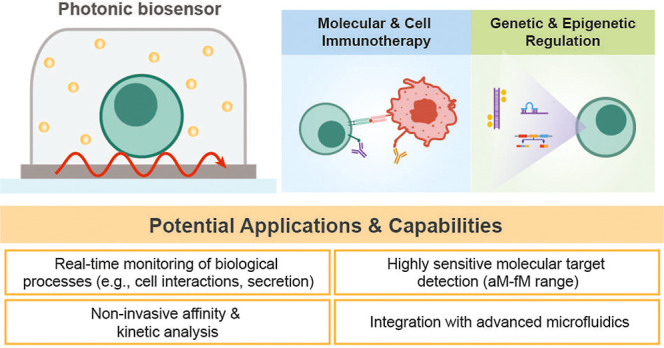

## Introduction

Cancer therapy is undergoing a profound transformation driven by advances in the deep knowledge of immunology, genomics, and cellular biology. Immunotherapies are reshaping the therapeutic landscape by harnessing the patient’s immune system to specifically recognize and eliminate malignant cells. This broad class of treatments encompasses molecular immunomodulators, adoptive cell transfer (ACT) strategies, and emerging gene- and epigenetic-regulation approaches, offering unprecedented precision in targeting tumor-specific cellular pathways (Figure 1). By integration of these modalities, immunotherapies have the potential to enhance therapeutic efficacy, improve the durability of clinical responses, reduce systemic toxicity, and enable personalized treatment regimens tailored to individual patient profiles.

**Figure 1:**
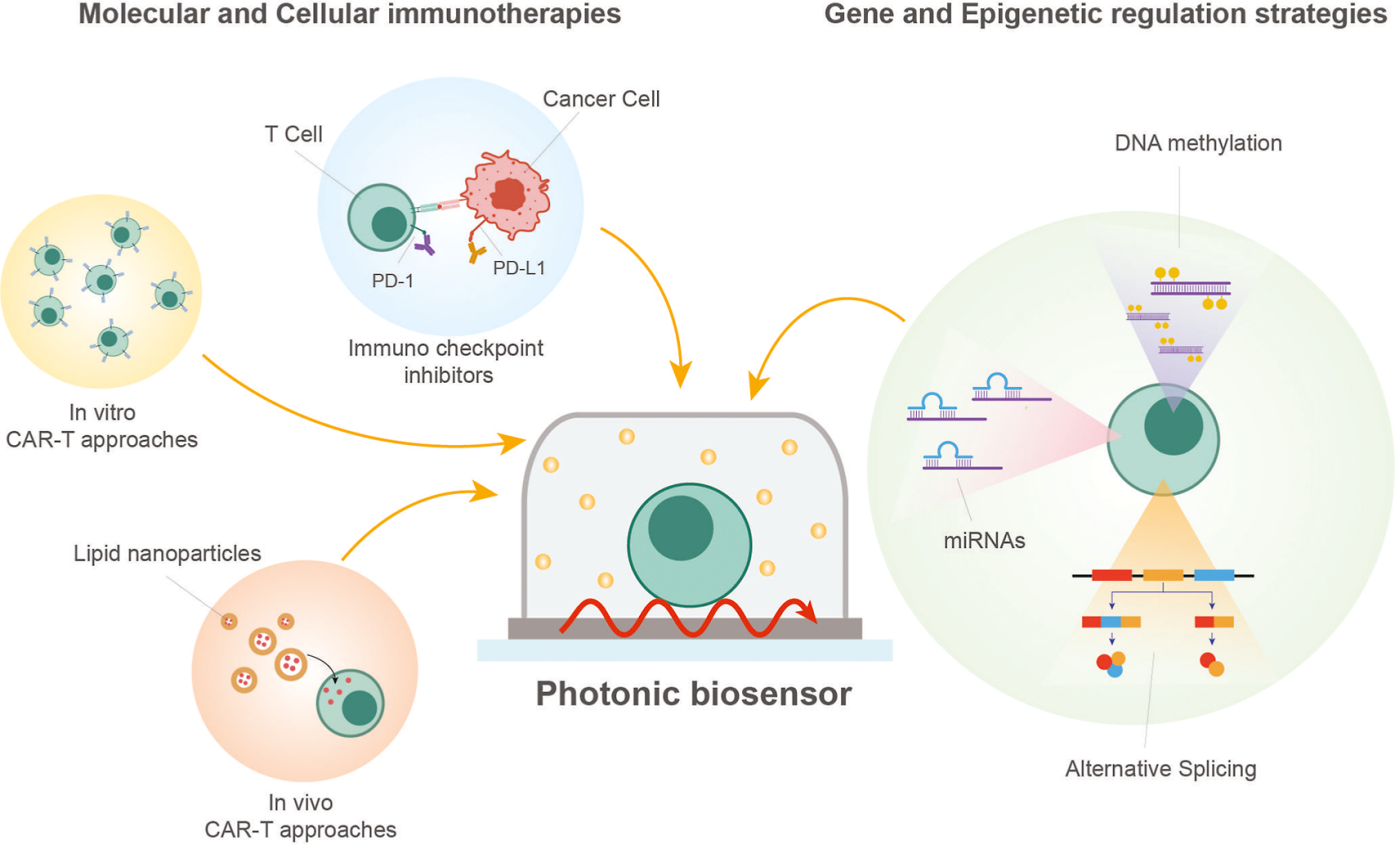
Schematic summary of key immunotherapy and cell-engineering modalities (i.e., molecular checkpoint inhibition, in vitro and in vivo CAR-T production routes, and gene/epigenetic regulation targets) that can be interrogated using label-free photonic biosensors for mechanistic studies and functional assessment.

Molecular immunotherapies, including immune checkpoint inhibitors (ICIs) targeting programmed cell death protein 1 (PD-1), programmed death-ligand 1 (PD-L1), and cytotoxic T-lymphocyte-associated antigen 4 (CTLA-4), together with tumor-directed monoclonal antibodies, have reshaped clinical oncology by restoring immune surveillance and inducing durable responses across multiple malignancies.^1,2^ In parallel, cell immunotherapies, and in particular chimeric antigen receptor (CAR)-T cells, have demonstrated remarkable clinical success in hematologic malignancies, producing high rates of durable remission in relapsed or refractory patients.^3^ Other ACT strategies, including T-cell receptor (TCR), tumor-infiltrating lymphocyte, and natural killer cell therapies, offer additional promise, particularly for solid tumors, as they can target a broader range of tumor-associated antigens (TAAs), exploit endogenous T-cell specificity, and harness innate cytotoxicity to overcome the challenges of heterogeneous and immunosuppressive tumor microenvironments.^4–6^ In addition, highly innovative in vivo CAR-T approaches using lipid nanoparticles or viral vectors are emerging as a transformative strategy to generate functional immune responses directly within patients, potentially providing scalable and more accessible alternatives to ex vivo manufacturing.^7^

Complementing these advances, interventions targeting genetic and epigenetic regulation, such as DNA methylation, alternative splicing (AS), and noncoding RNAs, provide powerful tools to further modulate both tumor and immune cell function.^8,9^ Epigenetic interventions, including DNA methyltransferase inhibitors and chromatin-modifying drugs, can restore the expression of silenced tumor suppressor genes and enhance immune cell activity.^10^ Splicing modulators and antisense approaches allow correction of aberrant isoforms that drive tumor progression or mediate immune evasion, while RNA-based therapeutics, including microRNA and long noncoding RNA regulators, can fine-tune key signaling pathways in both cancer and immune cells.^11–13^ By enabling precise, multilayered control over gene expression and cellular behavior, these gene-regulatory strategies can enhance the potency, specificity, and durability of immunotherapies. Together, the integration of molecular-, cellular-, and gene-regulation-based approaches defines a new therapeutic paradigm that could substantially increase the range of treatable tumors and improve patient outcomes.

Despite their transformative potential, these therapeutic modalities face major scientific and translational hurdles. Immune-related adverse events, tumor heterogeneity, and the immunosuppressive tumor microenvironment remain significant barriers to consistent efficacy, particularly in solid tumors. Moreover, challenges related to off-target effects, gene-editing safety, and the high cost and complexity of manufacturing cell-based products continue to limit their broad clinical implementation. Addressing these limitations requires an integrated framework that combines biological innovation with advanced analytical technologies capable of providing real-time, quantitative, and mechanistic insights into cellular and molecular dynamics.

The development and optimization of next-generation cancer therapies depend critically on analytical tools that can monitor treatment-induced changes in cell phenotypes, signaling networks, and gene expression with high spatial and temporal resolution. Traditional analytical techniques, such as flow cytometry, fluorescence, and confocal microscopy and molecular assays like ELISA or Western blotting, remain essential for characterizing immune cell populations, visualizing cellular interactions, and quantifying secreted factors. However, these methods typically provide population-averaged or end-point readouts, which makes it difficult to track the temporal evolution of individual cell states or capture the heterogeneity that emerges within phenotypically similar subsets. In addition, most of these techniques require fluorescent or biochemical labels, which can constrain the number of biomarkers detected simultaneously and frequently measured across separate assays or sample aliquots, preventing their direct correlation within the same single cell and further limiting our ability to map coordinated molecular changes. On the other hand, next-generation sequencing and epigenetic assays, including chromatin immunoprecipitation (ChIP) sequencing and bisulfite sequencing, have revolutionized our understanding of cellular heterogeneity and gene regulation, yet their high cost, technical complexity, and lack of real-time capability constrain their use in dynamic therapeutic monitoring.

To overcome these analytical barriers, label-free photonic biosensors based on the evanescent field sensing principle can become powerful tools to investigate molecular interactions and cellular processes in real time and under physiologically relevant conditions.^14^ By detecting subtle changes in optical properties such as refractive index, intensity, or resonance wavelength within the near-surface region of confined optical modes, these platforms enable noninvasive, continuous monitoring of biomolecular events without the need for fluorescent or radioactive labels. Plasmonic and integrated silicon photonic biosensors, in particular, combine high surface sensitivity with compatibility with microfluidic integration, miniaturization, and multiplexed analysis, making them especially suited for translational applications in cancer research.

In this perspective, we discuss the emerging role of evanescent wave-based photonic biosensors, with a specific focus on plasmonic and integrated silicon photonic platforms, as enabling technologies in the design and evaluation of advanced cancer immunotherapies. We highlight how these platforms can bridge the current analytical gap between molecular understanding and therapeutic translation by enabling real-time, quantitative assessment of immune and cancer cell regulation at the molecular and cellular levels. In addition, we critically examine the main technological and translational bottlenecks that currently limit the broader adoption of these optical biosensors and outline emerging strategies and research directions to address these challenges. Ultimately, such advances are expected to accelerate the rational design of safer, more effective, and truly personalized cancer immunotherapies.

## Evanescent Wave-Based Photonic Biosensors in Biomedicines

Photonic biosensors based on the evanescent wave principle have become indispensable analytical platforms in biomedical research, enabling label-free, quantitative, and noninvasive monitoring of biomolecular interactions. These devices integrate a biorecognition element, such as an antibody or nucleic acid probe, with an optical transducer that converts specific biological interactions into measurable optical signals (Figure 2A). By detecting variations in light properties within the evanescent field region, they allow for the direct identification and quantification of analytes without the need for fluorescent or colorimetric labels, removing the dependency on fluorophores and the multiplexing constraints associated with their limited spectral bandwidth. In these platforms, sensing relies on the interaction between light confined within a nanostructure or waveguide and the surrounding medium, where an evanescent electromagnetic field, extending several hundred nanometers beyond the sensor surface, responds sensitively to local refractive index changes upon analyte binding. This shift alters the propagation or resonance conditions of the optical mode, providing a direct and quantitative measure of biomolecular events. Among these technologies, plasmonic and integrated silicon photonic biosensors represent the most mature and widely adopted platforms, offering high sensitivity, versatility across a wide range of targets, and strong potential for multiplexing and integration into compact, user-friendly analytical devices.

**Figure 2:**
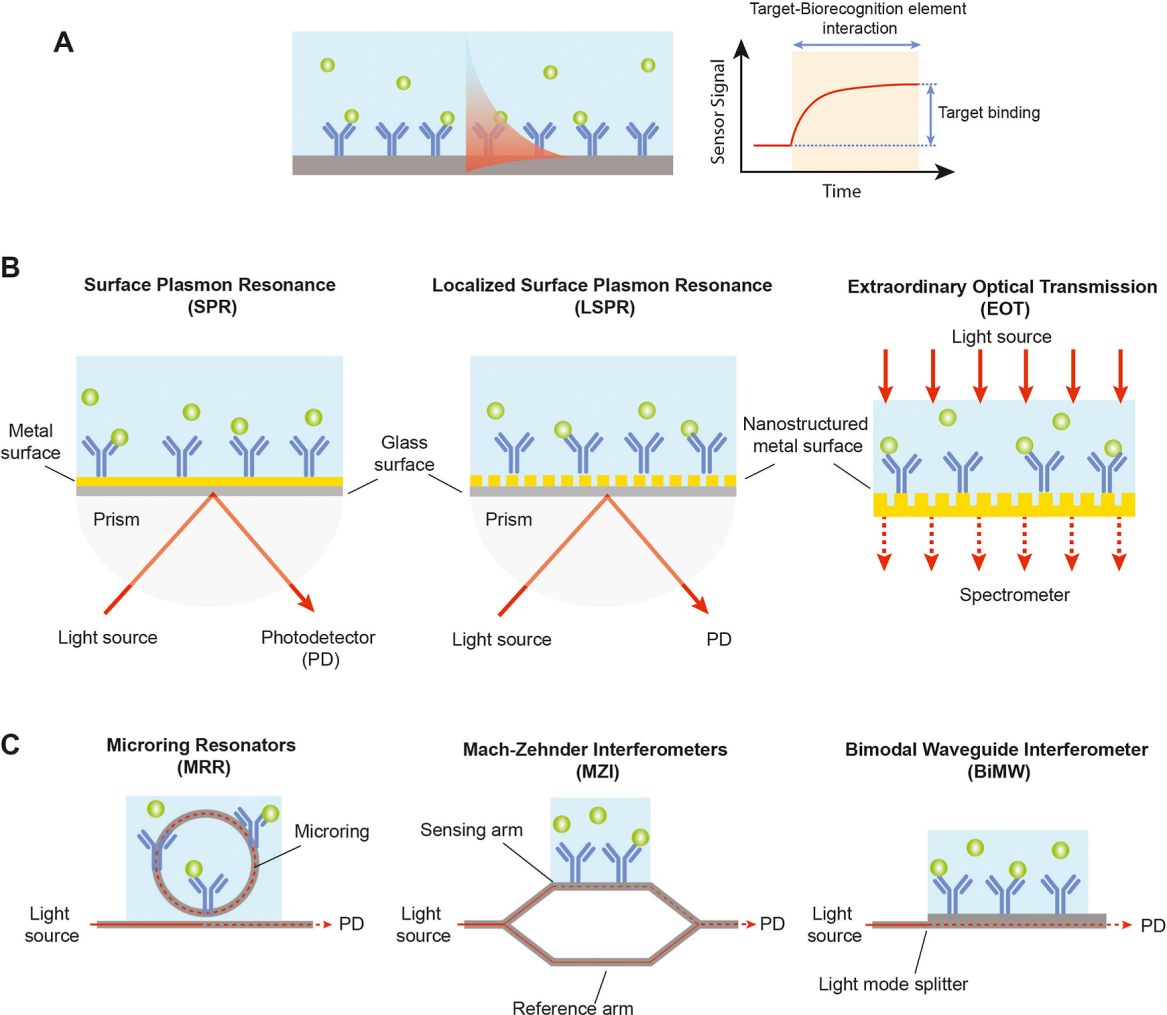
Schematic illustrations of label-free photonic sensor technologies and working principles: (A) generic photonic biosensor based on the evanescent field sensing principle and typical real-time sensorgram for the analysis of target–receptor interaction and quantification of target binding; (B) representative nanoplasmonic sensor configurations based on propagating surface plasmon resonance (SPR) (left), localized surface plasmon resonance (LSPR) (middle), which can be interrogated similar to SPR or via alternative optical readouts like transmission or scattering for enhanced integration (not shown in the figure), and extraordinary optical transmission (EOT); (C) representative waveguide-based silicon photonic sensor configurations based on microring resonators (miRR) (left), Mach–Zehnder interferometers (MZI) (middle), and bimodal waveguide (BiMW) interferometers (right).

Plasmonic biosensors, and in particular SPR systems, have long been the benchmark in label-free biomolecular analysis, with mature commercial platforms demonstrating their robustness, reproducibility, and quantitative capabilities. SPR relies on detecting refractive index changes at a metal–dielectric interface, typically by monitoring variations in the intensity or angle of the reflected light, which allows precise quantification of biomolecular binding kinetics and affinities (Figure 2B). This robust and well-standardized workflow has made SPR a cornerstone in pharmaceutical and biomedical research, with established applications in drug discovery, target validation, and quality control.^15^ Recent advances in nanoplasmonic architectures, including nanoparticle arrays and nanohole grids, have expanded SPR sensing toward LSPR and EOT modalities.^16^ These nanostructured platforms preserve evanescent-field sensing while enabling alternative optical readouts, facilitating integration into compact or on-chip formats. Such developments have pushed detection limits toward the single-molecule regime and enabled multiplexed analysis in miniaturized systems. Nevertheless, the need for sophisticated nanofabrication, precise biofunctionalization, and seamless integration of optical and microfluidic components continues to pose challenges for large-scale clinical deployment.

In parallel, integrated silicon photonic biosensors have emerged as a powerful alternative, combining high analytical performance with the scalability of semiconductor manufacturing.^17^ Devices based on ring resonators, Mach–Zehnder interferometers (MZI), and BiMW interferometers exploit the optical properties of silicon waveguide materials to achieve the sensitive detection of refractive index variations induced by biomolecular binding (Figure 2C). In resonant systems, these interactions lead to measurable shifts in the resonance wavelength, while in interferometric devices, phase changes alter the interference pattern. The integrated nature of these silicon photonic platforms enables dense multiplexing, compact footprints, and compatibility with standard complementary metal-oxide-semiconductor (CMOS) fabrication, supporting large-scale manufacturing and system integration. These attributes position silicon photonic biosensors as attractive candidates for next-generation biomedical and diagnostic platforms. Nonetheless, practical challenges remain, particularly in integrating sample handling, automation, and user-friendly interfaces, to translate laboratory prototypes into clinically validated bioanalytical tools.

In the last decades, research on evanescent wave-based photonic biosensors has increasingly focused on their application and validation in clinical diagnostics, targeting a broad range of analytes relevant to precision medicine. These include conventional and emerging cancer biomarkers (such as proteins, antibodies, microRNAs, and DNA mutations), infectious pathogens (viruses and bacteria), and therapeutic drug monitoring for personalized patient follow-up.^14^ A critical challenge in these applications is the control of nonspecific adsorption and background signals arising from complex biological matrices, such as serum, plasma, or blood, which can compromise specificity despite high intrinsic sensitivity. To address this issue, substantial advances have been made in surface biofunctionalization strategies, including the use of oriented and high-density capture layers, optimized linker chemistries, and affinity probes with improved selectivity, as well as in the development of antifouling coatings based on polymer brushes, zwitterionic materials, and mixed self-assembled monolayers.^18^ These approaches have enabled direct or minimally diluted analysis of complex biological fluids while preserving the advantages of real-time, label-free detection. Across numerous small-scale clinical validation studies, label-free biosensors have frequently demonstrated equivalent or superior analytical performance compared to traditional diagnostic assays, including ELISA, chemiluminescence immunoassays (CLIA), and polymerase chain reaction tests.^19^

Despite these advances, adapting these biosensors to the study of advanced therapies remains highly challenging due to the complexity and dynamic nature of living systems. Cell-based analyses involve multifactorial and transient processes, such as receptor signaling, morphological adaptation, and epigenetic modulation, that occur across multiple spatial and temporal scales. Capturing these subtle events requires biosensors with exceptional sensitivity and specificity, sufficient to enable fully direct, label-free detection without the need for secondary antibodies or sandwich assays while ensuring compatibility with physiologically relevant environments and maintaining cell viability. The need for multiplexed detection further increases system complexity as advanced therapies often engage multiple targets and pathways simultaneously. Integration with microfluidic platforms offers a promising path forward, enabling precise control of cell culture conditions, automated delivery of stimuli, and high-throughput, multidimensional measurements. Such convergence of label-free photonic sensing and microfluidic technology provides a powerful framework for real-time functional analysis of immune or tumor cells, bridging molecular detection with the evaluation of therapeutic efficacy in next-generation cancer treatments. In the following sections, we highlight representative biosensor platforms and methodologies with potential relevance to the design and evaluation of advanced cancer therapies, emphasizing their key advantages, current limitations, and future perspectives.

## Applications in Molecular Immunotherapies

The clinical success of ICIs and monoclonal antibodies (mAbs) has positioned these molecular immunotherapies as well-established, routinely administered cancer treatments. By targeting regulatory molecules such as PD-1, PD-L1, and CTLA-4, checkpoint inhibitors restore antitumor immune activity and have become standard-of-care across multiple malignancies, including melanoma, lung, renal, and urothelial cancers. Similarly, mAbs directed against TAAs, such as rituximab and trastuzumab, mediate direct cytotoxicity and can synergize with chemotherapy or targeted agents to improve efficacy while reducing systemic toxicity. Despite these achievements, challenges remain in extending these therapies to broader tumor types, optimizing combination strategies, and overcoming resistance mechanisms that limit durable responses.

In the design and development of these therapies, two main factors must be considered: the profiling of TAAs and immune checkpoint expression in either tumor or immune cells and the affinity and specificity of the therapeutic molecules toward the targeted receptor. The latter has been extensively addressed with diverse types of evanescent wave-based photonic biosensors by implementing simple biomolecular interaction assays for the screening of different candidates, identifying specific epitopes, and evaluating the interaction dynamics. These assays typically provide quantitative binding parameters, such as association (*k*_on_) and dissociation (*k*_off_) rates and equilibrium dissociation constants (*K*_D_), which are essential for ranking therapeutic candidates based on affinity and expected in vivo performance. For example, high-throughput SPR platforms have been used to generate epitope community maps integrated with affinity data across panels of therapeutic mAbs, enabling the identification of high-affinity lead candidates.^20^ Puopolo et al. also reported a protocol for the implementation in commercial SPR instruments to evaluate the effect of small molecules as PD1/PD-L1 checkpoint inhibitors.^21^ They proved comparable results to standard techniques like ELISA or cell-based assays (IC_50_ values around 85 and 650 nM) with shorter experimental runs and lower sample volume requirements. Nonetheless, interesting approaches are also being developed to enhance the biomimetic characteristics of the sensor assay. In this regard, Batool et al. published an innovative biomimetic sensor based on nanoplasmonic arrays coated with a functionalized lipid bilayer, simulating an artificial tumor cell membrane expressing PD-L1 molecules (Figure 3A).^22^ They demonstrated high sensitivity for the monitoring of PD-L1/PD-1 interactions, achieving an LOD of 6.7 ng/mL (0.2 nM) as well as accurate evaluation of specific mAbs as checkpoint inhibitors (IC_50_ = 0.43 nM). Unlike standard surface chemistry procedures for bioreceptor immobilization, the functional lipid membrane enables lateral diffusion of the attached receptors, facilitating the formation of cell-interaction clusters. Another example is the work from Álvarez Freile et al., who utilized spherical optical resonators coated with specific tumor receptors (Epidermal Growth Factor Receptor, EGFR) and immune checkpoints (PD-L1) to imitate a cancer cell and monitor the interaction with specific therapeutic mAbs (i.e., cetuximab for EGFR and atezolizumab for PD-L1) (Figure 3B).^23^ Their biosensor offers a versatile platform for the screening and evaluation of different molecular immunotherapies in a three-dimensional (3D) assay format.

**Figure 3:**
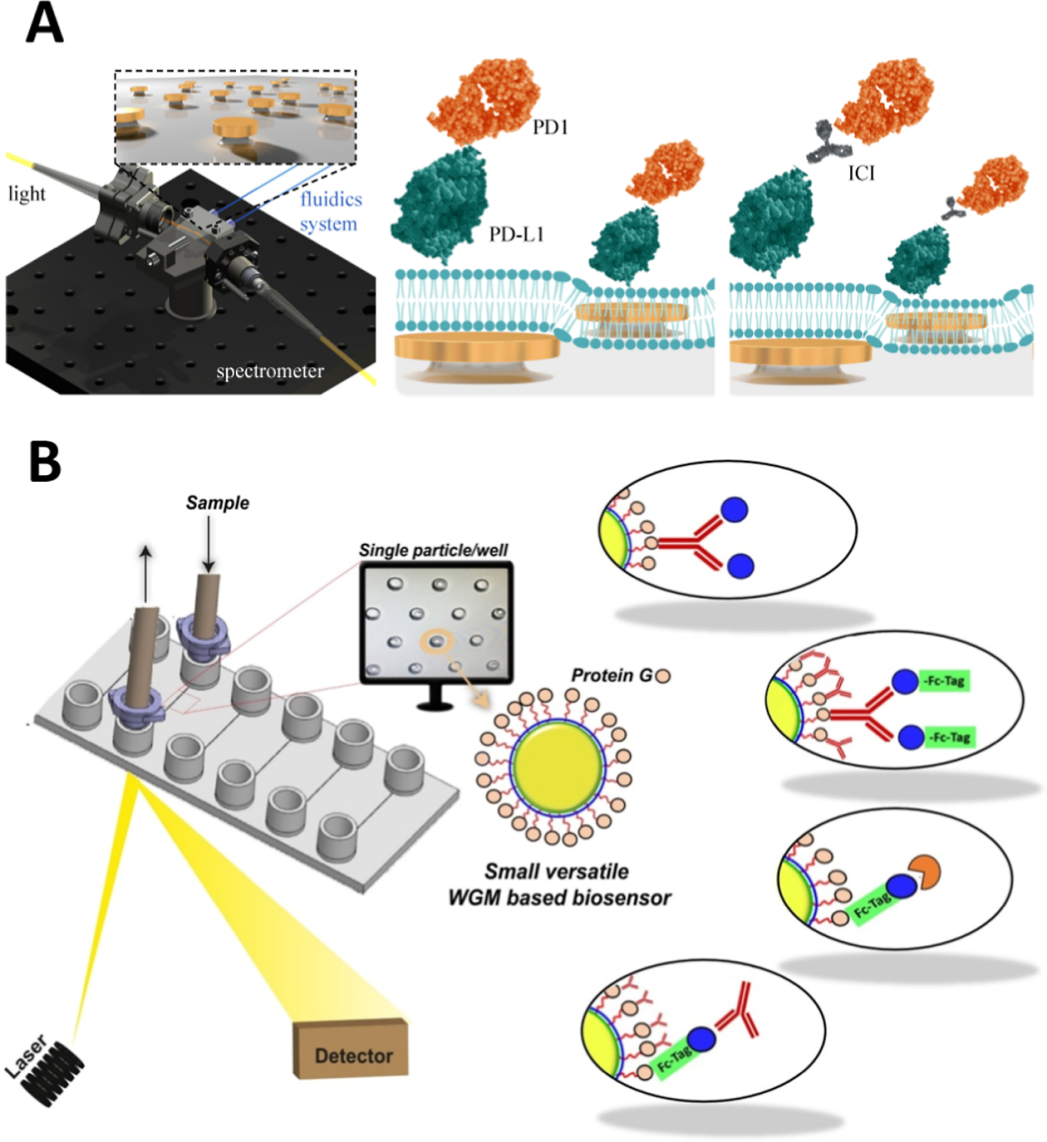
Examples of evanescent wave-based biosensors applied in molecular immunotherapy evaluation: (A) plasmonic nanodisk arrays coated with lipid membranes for monitoring interactions of checkpoint inhibitors. Reproduced from ref 22 under the terms of the Creative Commons license (CC-BY), published by Springer Nature. Copyright 2024 The Authors; (B) spherical optical resonators coated with tumor antigens and immune checkpoints to monitor interaction with therapeutic antibodies. Reproduced from ref 23 under the terms of the Creative Commons license (CC-BY), published by Elsevier B.V. Copyright 2021 The Authors.

Conversely, there is currently very little research focused on the study and identification of tumor antigens or immune receptors directly in cells using evanescent wave-based photonic biosensing. Despite numerous studies having been published for the detection of soluble markers, exosomes, or circulating tumor cells, the design and evaluation of effective immunotherapies critically require an accurate profiling of the expression of such molecules within the tumor microenvironment. This study is commonly performed with fluorescence-based methods, and it would greatly benefit from a highly sensitive technique that allows for fast detection and quantification in a noninvasive and multiplexed format. In this direction, Shanehbandi et al. proposed a SPR immunosensor for the identification of CD20-positive lymphoma cells through their interaction with specific antibodies immobilized on the surface.^24^ In this proof-of-concept study, SPR signals correlated with the density of CD20 expression on cells, and the biosensor was able to distinguish CD20-positive from CD20-negative cells with a LOD of around 10^4^ cells/mL, demonstrating feasibility for direct cellular receptor profiling. It represents a proof of concept of the potential use of SPR biosensors in the analysis of cell receptor expression. A more advanced study has recently been published by Ahmed et al., introducing a plasmonic-based surface-enhanced Raman scattering (SERS) technology for deciphering the heterogeneity of diverse immune checkpoints expression in lung cancer cells.^25^ This mesoporous gold SERS platform enabled label-free spectral discrimination of immune checkpoint expression at the single-cell level, quantifying differential expression patterns across individual cells within patient samples and revealing inter- and intratumoral heterogeneity; the authors reported statistically significant differences in SERS signal intensities corresponding to variable levels of checkpoint proteins such as PD-L1 and CTLA-4. These examples underscore how quantitative cell-level measurements of antigen or receptor expression could inform the rational design and personalized selection of immunotherapies.

Future efforts should aim to translate optical biosensing from simplified biomolecular assays to more physiologically relevant models. The integration of biomimetic interfaces, multiplexed detection, and microfluidic handling will be key to capturing the dynamic and heterogeneous interactions that define the tumor–immune microenvironment. Combining these technologies with single-cell or patient-derived analyses could enable the rapid and quantitative profiling of immune checkpoints and tumor antigens, providing actionable insights for the design of tailored molecular immunotherapies.

## Applications in Cell Immunotherapies

The clinical success of CAR-T cell therapies in hematologic malignancies has driven widespread interest in ACT approaches as a groundbreaking strategy for cancer immunotherapy. Currently, these therapies rely on the ex vivo engineering or expansion of patient-derived immune cells, typically T lymphocytes, to recognize and eradicate tumor cells through antigen-specific mechanisms. Upon reinfusion, the modified cells can proliferate, persist, and mediate durable antitumor responses, as demonstrated by the impressive remission rates achieved in B-cell leukemias and lymphomas. Despite these breakthroughs, translating ACT therapies to solid tumors remains a major challenge due to the high heterogeneity and complexity of the tumor microenvironment, which includes immunosuppressive signaling, physical barriers that limit immune-cell infiltration, and dynamic antigen loss or modulation that favors immune evasion.

Here, the role of evanescent wave-based photonic biosensors can be directed to facilitate and improve both the analysis of immune-tumor cell interactions and the evaluation of effective activation of immune response mechanisms in different tumor environments. An important limitation in studying cell–cell binding dynamics with these technologies is their on-surface sensing mechanism, which does not allow for probing interactions occurring farther than 300–500 nm from the sensor surface. Nonetheless, strategies can be developed to functionalize the sensor surface with specific tumor ligands and analyze the interaction with immune cells. In this sense, an SPR biosensor coated with an artificial cell membrane expressing TAAs (through a recombinant peptide Major Histocompatibility Complex—pMHC) was applied for the quantitative, two-dimensional affinity analysis of tumor-specific T-cell receptors (TCRs) (Figure 4A).^26^ The platform discriminated high-affinity from low-affinity TCRs with picomolar-to-nanomolar effective affinity differences, showing a strong correlation with flow cytometry while providing higher sensitivity and improved resolution compared to conventional SPR assays using immobilized proteins.

**Figure 4:**
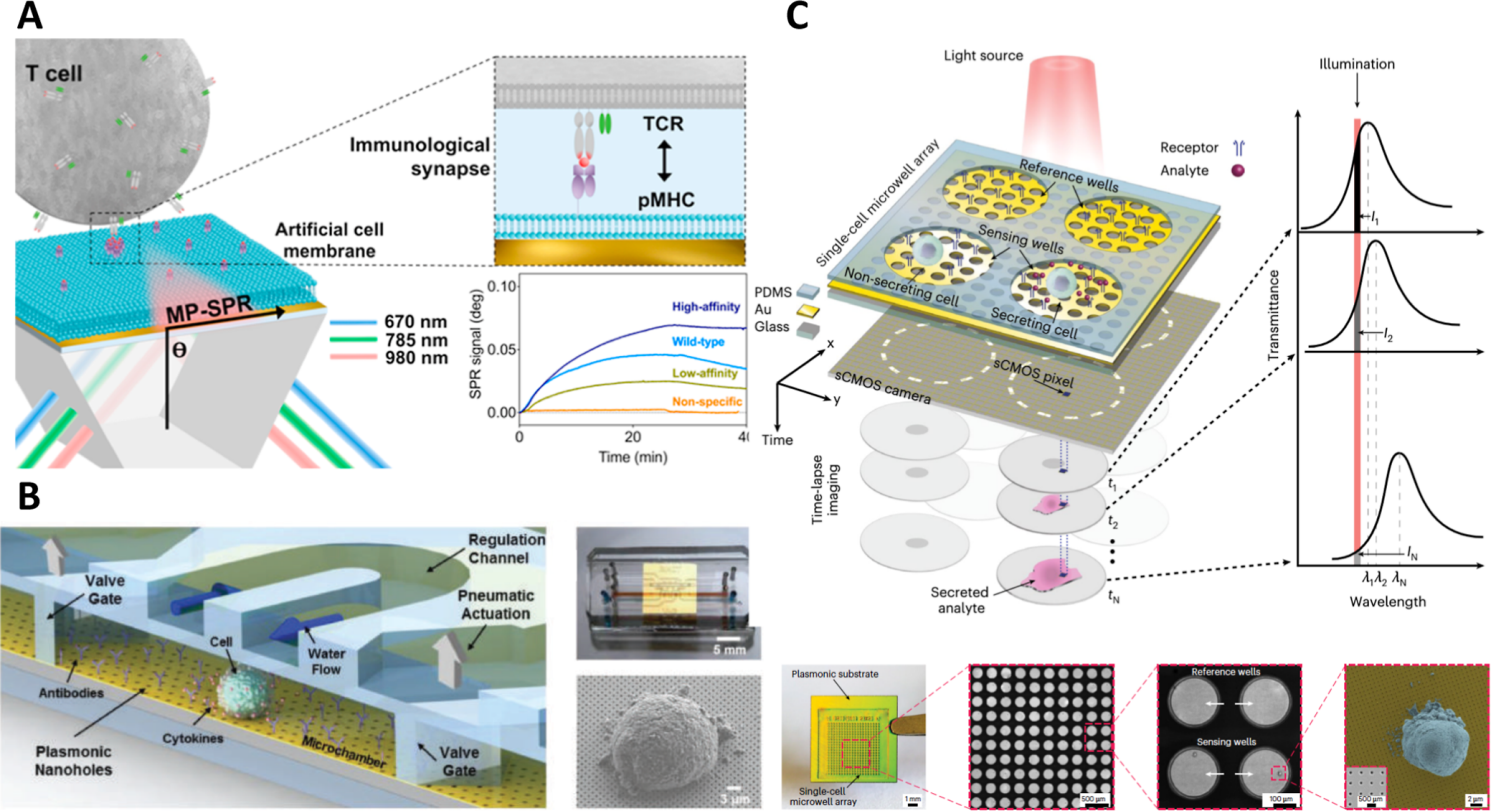
Examples of label-free optical biosensor applied in cell immunotherapy evaluation: (A) plasmonic biosensor coated with tumor-mimicking membranes to analyze avidity and specificity of engineered T cells. Reproduced from ref 26. Copyright 2018 American Chemical Society; (B) plasmonic nanohole array sensor integrated with advanced microfluidic systems for monitoring of cytokine secretion at the single-cell level. Reprinted in part from ref 27. Copyright 2018 Wiley-VCH; (C) plasmonic nanohole array sensor integrated with the microwell system for high-throughput single-cell secretion monitoring. Reprinted in part from ref 29 under the terms of the Creative Commons license (CC-BY), published by Springer Nature. Copyright 2023 The Authors.

For the analysis of immune activity and signaling events, immunoassay-based techniques such as ELISpot and FluoroSpot have become valuable tools, providing secretion detection and functional assessment at single-cell resolution. However, these assays are limited by end point measurements, labor-intensive protocols, and the inability to recover live cells for downstream analyses, which restricts real-time monitoring and longitudinal studies of the same cells. To address these limitations, plasmonic biosensors have been increasingly applied to study immune cell secretion and signaling with high sensitivity and temporal resolution. Li et al. developed an optofluidic nanoplasmonic sensor capable of real-time quantification of cytokine secretion at the single-cell level, achieving detection limits in the low pg/mL range and temporal resolution on the order of seconds (Figure 4B).^27^ This concept was later expanded from a single-plex to a high-throughput format through integration with microwell microfluidics (Figure 4C).^28,29^ These platforms revealed pronounced functional heterogeneity in immune cell populations, including asynchronous secretion onset times, burst-like release patterns, and cell-to-cell variability in cumulative cytokine output, features that are inaccessible to bulk or end point assays. Similarly, Juan-Colás et al. employed resonant photonic crystal sensors combined with hyperspectral imaging to achieve single-cell cytokine mapping, providing quantitative measurements alongside spatial distribution profiles.^30^ Despite these advances, label-free approaches capable of simultaneously detecting a broad panel of cytokines relevant to antitumor immune responses are still lacking, highlighting an important opportunity for biosensor technologies in immunotherapy development.

In addition, these technologies could support the development of novel in vivo CAR-T strategies, such as lipid-nanoparticle-mediated delivery, by enabling early functional assessment of T-cell activation, antigen recognition, and preliminary safety evaluation in biomimetic models. By providing quantitative, real-time readouts of cytokine secretion, receptor engagement, and cell–cell interactions, evanescent wave-based photonic biosensors can reveal critical information about CAR-T behavior before in vivo administration. Such applications may help optimize delivery systems, guide CAR design to enhance efficacy and specificity, and detect potential off-target or overstimulation effects early in the development pipeline. Furthermore, coupling these biosensors with high-throughput or multiplexed platforms could accelerate iterative testing of multiple CAR constructs, nanoparticle formulations, or costimulatory signals, providing rapid feedback on therapeutic potential and informing rational decisions prior to clinical translation.

Taken together, evanescent wave-based photonic biosensors offer a versatile platform for improving the analysis, optimization, and evaluation of cell immunotherapies, bridging the gap between experimental studies and clinical translation. Looking ahead, research should focus on expanding the capabilities of these platforms to more comprehensively model and monitor complex tumor–immune interactions. Strategies to overcome the spatial limitations of surface-based sensing, such as three-dimensional biomimetic scaffolds, microfluidic cell coculture systems, or engineered extracellular matrices, could enable the study of immune-cell infiltration and synapse formation in physiologically relevant contexts. In parallel, the development of multiplexed biosensing platforms capable of simultaneously tracking multiple cytokines, immune checkpoints, and receptor–ligand interactions at the single-cell level will be critical for capturing the dynamic heterogeneity of antitumor immune responses. Ultimately, advancing label-free biosensor technologies in these directions has the potential to accelerate the rational design, evaluation, and clinical translation of next-generation cell immunotherapies while providing deeper mechanistic insights into immune function and tumor biology.

## Applications in Gene Regulation Strategies

Gene regulation at multiple levels, including epigenetic modifications, chromatin accessibility, AS, and noncoding RNA regulation, is emerging as a critical determinant in cancer progression, immune cell function, and therapeutic response. Dysregulation in these pathways contributes to tumor heterogeneity, therapy resistance, and the variable efficacy of immunotherapies. Epigenetic alterations, such as DNA methylation, can influence the expression of key immune checkpoints and the activity of CAR-T cells.^31,32^ Hypermethylation of promoter regions in tumor cells often leads to the silencing of antigen-processing machinery and downregulation of TAAs, reducing immune recognition. Conversely, hypomethylation within regulatory regions of genes such as PD-L1 can increase checkpoint ligand expression, driving T-cell exhaustion and enabling immune escape. In engineered immune cells, targeted modulation of methylation states has been shown to stabilize effector phenotypes, enhance memory formation, and reduce the acquisition of exhaustion-associated transcriptional programs, thereby improving the in vivo persistence of CAR-T cells. Aberrant splicing and noncoding RNA expression further modulate both tumor and immune cell behavior.^33,34^ For example, AS generates isoforms of PD-1, CTLA-4, CD28, and CD19, with distinct signaling capacities, some of which can attenuate T-cell activation or enable tumor immune evasion. Similarly, modulating splicing factors such as SRSF2 or hnRNPA1 can tune cytokine production, exhaustion profiles, and metabolic fitness of T cells. Several microRNAs, such as miR-155 or miR-17–92, can enhance T-cell activation, proliferation, and resistance to exhaustion, while long noncoding RNAs regulate chromatin accessibility and transcriptional programs tied to cytotoxicity memory formation or tumor-induced dysfunction. Manipulating these DNA- and RNA-based regulatory networks through genetic editing, epigenetic drugs, or delivery of synthetic RNA modulators offers complementary strategies to boost the therapeutic efficacy, durability, and resilience of immunotherapies. Therefore, analyzing and monitoring these dynamic regulatory events in real time could provide valuable insights for the design, evaluation, and optimization of next-generation cancer therapies.

Evanescent wave-based photonic biosensors have been demonstrated as versatile tools for detecting DNA methylation and balancing sensitivity, specificity, and sample preparation requirements. For example, Yoon et al. integrated bisulfite conversion, amplification, and detection into a lab-on-a-chip platform with microring resonators, enabling rapid and sensitive detection of as little as 1% methylated DNA in mixed samples and input DNA amounts in the low nanogram range (Figure 5A).^35^ An alternative strategy that bypasses bisulfite conversion is the use of antibody-based biosensors, which enable rapid detection by directly capturing methylated cytosines, typically achieving nM sensitivity, although they generally lack sequence specificity. Direct recognition of double-stranded DNA using triplex-forming hairpin probes or CpG-binding proteins has also been demonstrated, providing real-time, label-free quantification of methylated regions (Figure 5B).^36^ In particular, this biosensor enabled discrimination of methylated versus unmethylated DNA fragments with detection limits in the low nanomolar range and without enzymatic preprocessing while preserving sequence selectivity. In addition, optical biosensors can discriminate cytosine oxidation products, such as 5-hmC and 5-fC, through surface functionalization with selective antibodies or chemical receptors, with reported sensitivities down to the sub-nM range, enabling differentiation between closely related epigenetic modifications.^37^ These advances illustrate the potential of label-free optical platforms to provide rapid, sensitive, and increasingly sequence- or modification-specific DNA methylation analysis, paving the way for applications in cancer epigenetics and therapy monitoring.

**Figure 5:**
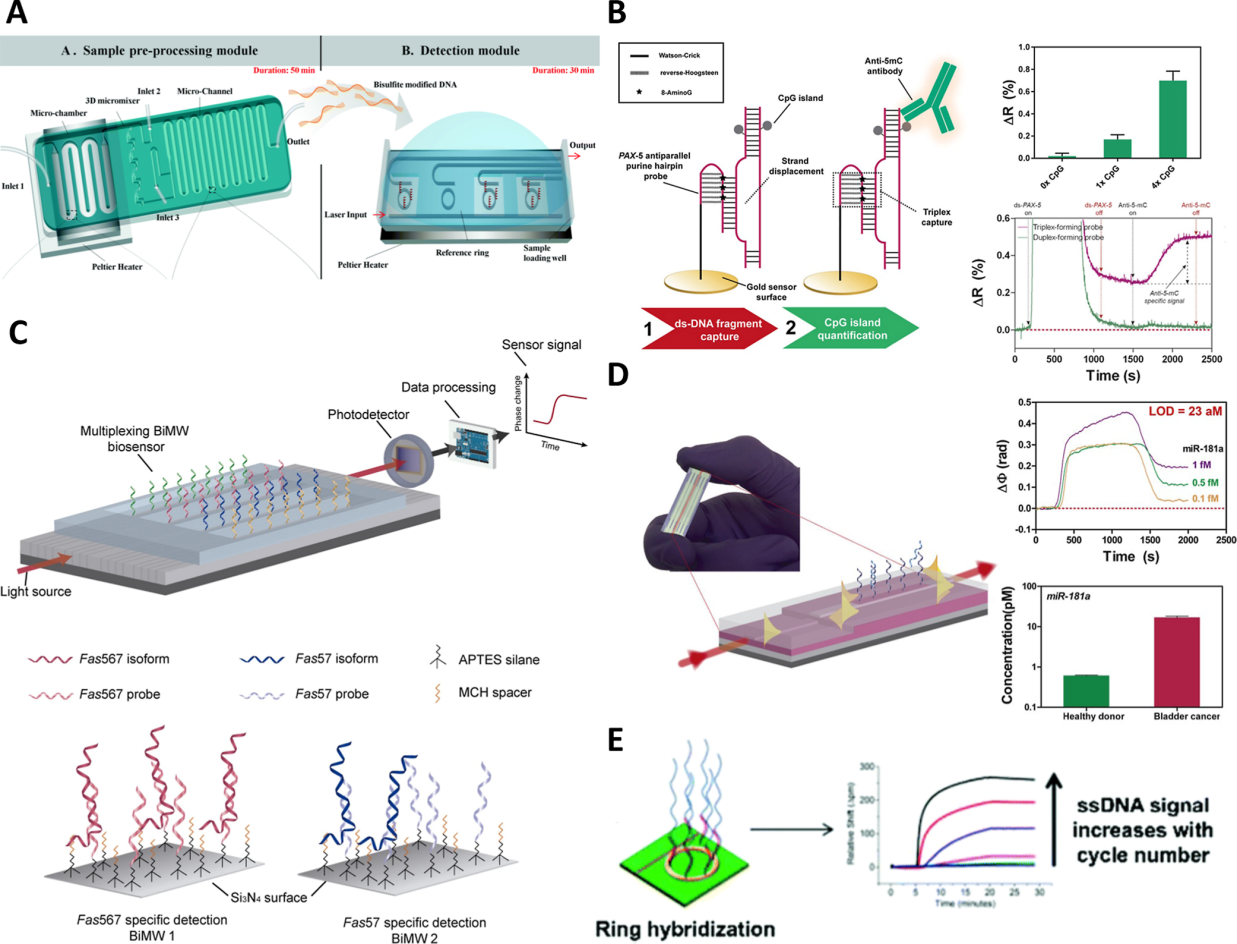
Examples of label-free optical biosensor applied in the analysis of epigenetic cell regulation: (A) microring resonators lab-on-a-chip platform for bisulfite conversion, DNA amplification, and DNA methylation analysis. Reprinted in part from ref 35. Copyright 2015 Royal Society of Chemistry; (B) plasmonic biosensor for quantification of DNA methylation in specific gene regions through triplex-forming hairpin probe capturing. Reproduced from ref 36. Copyright 2018 Elsevier B.V.; (C) multiplexed interferometric sensor for analysis of AS. Reprinted in part from ref 40 under the terms of the Creative Commons license (CC-BY), published by Springer Nature. Copyright 2017 The Authors; (D) BiMW sensor for ultrasensitive detection of microRNAs in biological samples. Reproduced from ref 42. Copyright 2016 American Chemical Society; (E) microring resonators for analysis of long noncoding RNAs. Reprinted in part from ref 44. Copyright 2018 Royal Society of Chemistry.

These optical biosensors can also be used for the study of AS events and their modulation in cancer and immunotherapy. AS regulates proteomic diversity and influences key immune and oncogenic pathways, making its precise monitoring crucial for understanding the therapy response. By facilitating rapid screening of splice-modulating drugs or CRISPR-based editing strategies, such platforms could provide actionable insights for optimizing therapeutic efficacy.^38^ Huertas et al. achieved highly selective detection of splice variants from the Fas and Bcl-x genes—key regulators of apoptosis—using different optical biosensors capable of distinguishing isoforms with negligible cross-reactivity (<5%), within 20 min, at pM to nM detection limits, depending on probe design and target length (Figure 5C).^39,40^ Precleaving long mRNA molecules before hybridization can further enhance accessibility and sensitivity, yielding up to 1 order of magnitude improvement in the signal-to-noise ratio and detection limits, making the performance comparable to that of standard PCR-based assays.^41^ Furthermore, the integration of photonic biosensors with microfluidic systems enables single-cell trapping and analysis, which is a highly relevant approach for studying AS dynamics in immune cells during immunotherapy.

Beyond epigenetic and splicing regulation, noncoding RNAs (ncRNAs) play a pivotal role in modulating immune cell differentiation, effector functions, and resistance to immunotherapy. Aberrant ncRNA expression can influence immune checkpoints, cytokine signaling, and the cytotoxic potential of T cells, thus affecting the outcome of treatments such as checkpoint blockade or CAR-T cell therapy. Therapeutic approaches targeting ncRNAs, such as using miRNA mimics to restore tumor-suppressive functions, antagomirs to silence oncogenic miRNAs, or engineering immune cells with defined ncRNA profiles, are being actively explored to enhance immune responses and overcome resistance. In this context, label-free photonic biosensors could be used to monitor ncRNA-mediated regulatory processes in real time, bridging the gap between the mechanistic understanding and therapeutic application. For example, integrated photonic devices such as BiMW, achieving attomolar detection limits for miRNAs directly in biofluids without labeling or amplification (Figure 5D),^42^ could be exploited to track miRNA signatures that modulate T-cell exhaustion, antigen presentation, or cytokine signaling during immunotherapy. Similarly, the direct, amplification-free detection of circulating miRNAs at fM to aM concentrations, as demonstrated by Calvo-Lozano et al., illustrates how these sensors could monitor therapy-induced molecular changes within analysis times below 30 min dynamically and noninvasively, supporting personalized treatment adjustment.^43^ The same sensing principles can be extended to long noncoding RNAs (lncRNAs), which are emerging as critical regulators of chromatin remodeling and immune checkpoint expression. Photonic biosensors employing microring resonators have already demonstrated multiplex detection of lncRNAs targets with pM sensitivity and high sequence selectivity in cancer models, underscoring their adaptability to complex RNA targets (Figure 5E).^44^ Integrating these sensing architectures with microfluidic or single-cell systems could allow time-resolved monitoring of ncRNA-driven transcriptional reprogramming in immune cells exposed to therapeutic stimuli or gene-editing interventions. Finally, the ability of optical biosensors to directly quantify exosomal ncRNAs in minimally processed samples could open new opportunities to study intercellular communication between tumors and immune cells. Since exosome-associated ncRNAs can mediate resistance to checkpoint blockade or modulate immune activation, real-time monitoring of their dynamics using photonic biosensors could provide functional insights inaccessible to conventional molecular assays. Overall, these advances highlight how label-free optical biosensing platforms can evolve from diagnostic tools to mechanistic probes, enabling the dynamic, high-resolution interrogation of ncRNA-mediated regulation in next-generation immunotherapies.

By providing a unified, label-free, and dynamic framework, optical biosensors allow comprehensive profiling of gene-regulatory states in tumor cells, engineered immune cells, or patient-derived samples, offering a direct link between molecular interventions and their functional impact on immune responses and guiding the development of more precise and effective immunotherapy strategies. Future research should focus on expanding evanescent wave-based photonic biosensors to enable multiplexed, single-cell monitoring of dynamic gene-regulatory events in tumor and immune cells. Integration with microfluidic, organoid, or patient-derived systems could provide real-time insights into how therapeutic interventions reshape cellular function and immune responses. Enhancing sensor sensitivity and specificity will allow simultaneous detection of multiple regulatory targets and functional readouts, such as cytokine secretion or T-cell activation, offering a holistic view of therapy efficacy and safety. These advances have the potential to accelerate the rational design, optimization, and personalization of next-generation immunotherapies through dynamic, mechanistic feedback.

## Major Challenges and Opportunities

While promising development has been made, the integration of plasmonic and silicon photonic biosensors into the study and evaluation of advanced immunotherapies remains at an early stage and is currently constrained by several technological and translational bottlenecks. At present, many of the reported applications remain proof-of-concept or early translational studies in which performance metrics are typically obtained under controlled or simplified experimental conditions; therefore, more extensive validation using clinically relevant samples and larger cohorts will be required to rigorously establish sensitivity, specificity, and robustness for routine biomedical implementation. Besides, the complexity of immunotherapeutic mechanisms, ranging from molecular checkpoint modulation to the reprogramming of immune and tumor cells through gene-regulatory interventions, poses significant technical and analytical challenges that must be addressed through targeted methodological and engineering advances for these platforms to reach their full potential.

Among the most critical bottlenecks for the practical deployment of evanescent wave-based photonic biosensors is their performance in complex biological samples. Biosensors must operate reliably in matrices such as blood, tumor interstitial fluid, or complex culture media where the abundance of nonspecific biomolecules can mask or distort the signal from target analytes. This is particularly problematic when studying low-abundance regulatory molecules such as specific splice variants, ncRNAs, or methylated DNA regions, which often require detection limits in the femto- to attomolar range. Maintaining such sensitivity and selectivity in clinically relevant samples remains a central challenge. At the same time, this challenge opens opportunities for the development of innovative surface chemistries, including antifouling polymer brushes, zwitterionic coatings, and biomimetic membranes that preserve sensor sensitivity while minimizing background noise.^18^ Coupling these with automated lab-on-a-chip modules for rapid sample preparation, such as on-chip plasma separation, nucleic acid extraction, or exosome isolation, could substantially enhance applicability.^45,46^ These strategies can preserve sensor specificity and sensitivity in complex biological fluids, enabling direct, label-free detection in a real-time format.

Another crucial limitation lies in multiplexing capability. Immunotherapies act on intricate molecular networks involving immune checkpoints, cytokines, transcriptional regulators, and epigenetic modifiers. Capturing this complexity requires the simultaneous detection of multiple molecular and cellular biomarkers in a single assay. Although photonic architectures such as microring resonators and bimodal interferometers have demonstrated multiplexed detection at the research level, expanding this capability to complex samples without cross-reactivity or signal interference is still technically demanding. Promising research directions include the use of advanced computational strategies based on wavelength division or spectral deconvolution to expand multiplex capacity without compromising sensitivity.^47,48^ Advances in integrated photonics, such as on-chip tunable lasers or broadband frequency combs, could further support parallel detection of large biomarker panels relevant to immune activity.^49,50^

Integration with cell-based and functional assays represents an additional major challenge and opportunity. The effectiveness of immunotherapies depends not only on molecular signatures but also on the dynamic behavior of immune cells—such as activation, exhaustion, or cytotoxic function—which evolve over time in response to therapeutic interventions. Combining label-free optical biosensing with high-throughput microfluidic cell-trapping arrays or lab-on-chip platforms could enable real-time monitoring of immune cell interactions, signaling events, and phenotypic transitions under controlled conditions, providing a direct link between molecular regulation and functional immune outcomes.^29^ However, realizing this potential requires overcoming substantial engineering and analytical challenges. Maintaining cell viability, ensuring precise analyte delivery, and achieving stable and reproducible optical readouts within complex microenvironments remain key obstacles. These issues also highlight opportunities for next-generation microfluidics, including single-cell encapsulation, dynamic perfusion systems, and programmable microvalve networks that precisely modulate the cellular microenvironment.^27,51,52^ Furthermore, dynamic and longitudinal monitoring of the therapy response introduces additional layers of complexity. Immunotherapies often induce time-dependent molecular and cellular adaptations, necessitating biosensors capable of continuous or repeated measurements over extended periods. Achieving this will depend on advances in sensor robustness, automated microfluidic control, and data acquisition systems with emerging opportunities in the development of self-calibrating optical architectures, photonic materials with improved thermal and mechanical stability, and microfluidic reservoirs that support long-term culture and stimulation cycles.

Additionally, the integration of 3D-printed microfluidics or organ-on-chip (OoC) modules could further enable more physiologically relevant in vitro models for evaluating immunotherapy performance.^53^ Moreover, following the recent FDA announcement formally recognizing organ-on-chip platforms as valid disease models for regulatory testing, the combination of OoC systems with integrated biosensing further underscores their potential to bridge the gap between conventional in vitro assays and clinical outcomes.^54^ These biosensors allow continuous, noninvasive monitoring of molecular and cellular events, such as cytokine secretion, receptor–ligand interactions, or immune cell infiltration, directly within the microfluidic compartments of the OoC system. By providing real-time data without the need for fluorescent labels, label-free biosensors can capture dynamic biological processes, link molecular signatures to functional outcomes, and facilitate mechanistic studies of therapeutic responses. This approach could accelerate preclinical testing, support the optimization of dosing and treatment schedules, and provide actionable insights for the rational design of next-generation immunotherapies under conditions that closely mimic human tissue physiology.

Equally important, and often underestimated as a bottleneck, is the need for advanced computational frameworks to interpret the vast data sets generated by multiplexed, time-resolved biosensing. These data sets, which capture kinetic binding events and cellular dynamics in real time, require sophisticated algorithms for noise filtering, kinetic modeling, and correlation with clinical or biological outcomes. The integration of artificial intelligence and machine learning could significantly enhance this process by automatically detecting subtle patterns and correlations across large multidimensional data sets, identifying predictive biomarkers of therapy response, and optimizing experimental parameters such as analyte concentrations or timing of interventions. AI-driven approaches can also support predictive modeling of immune dynamics, enabling simulation of therapeutic outcomes and guiding rational experimental design. Furthermore, by combining biosensor readouts with clinical and omics data, data-driven analytics can inform personalized treatment regimens, adapt dosing schedules in real time, and uncover novel mechanistic insights into tumor–immune interactions. Ultimately, the convergence of optical biosensing, microfluidics, and data-driven analytics holds great promise for developing adaptive, feedback-informed immunotherapies capable of responding to the evolving landscape of tumor–immune interactions in real time.

Beyond analytical performance, the broader adoption of evanescent wave-based photonic biosensors in cancer immunotherapy research will critically depend on progress in standardization, large-scale validation, and economic scalability. At present, the technological maturity of the different platforms remains heterogeneous. Classical SPR systems benefit from relatively established commercial instrumentation and partial standardization in biochemical analysis, yet their translation into routine clinical workflows remains limited. Emerging nanoplasmonic configurations offer enhanced miniaturization and multiplexing potential but frequently rely on custom nanofabrication processes and laboratory-specific optical setups that hinder interlaboratory reproducibility and cost comparability. A similar variability is observed in integrated silicon photonic technologies that, although compatible with CMOS manufacturing and theoretically amenable to large-scale production, are still predominantly implemented as research prototypes with diverse surface chemistries, coupling schemes, and microfluidic integrations. Consequently, reported fabrication costs, instrumentation requirements, and operational expenses vary widely and are rarely assessed through standardized technoeconomic analyses, making direct cross-platform comparisons premature. Equally important is the limited availability of multicenter validation studies using clinically relevant cohorts, as most reported applications remain as proof-of-concept demonstrations or small-scale laboratory investigations. Establishing shared reference materials, harmonized biofunctionalization protocols, interlaboratory benchmarking initiatives, and regulatory-aligned performance criteria will therefore be essential steps toward transforming these highly sensitive analytical technologies from promising experimental tools into robust and standardized platforms capable of supporting the development and optimization of next-generation cancer immunotherapies.

Overall, while evanescent wave-based photonic biosensors have already proven their sensitivity, specificity, and versatility for molecular detection, their broader application to immunotherapy evaluation and optimization is still limited by identifiable technological and translational bottlenecks and defines clear research directions for the field. Systematically addressing these challenges through coordinated advances in biofunctionalization, microfluidic and system integration, industrialization, and data-driven analytics will not only enhance our ability to study immune mechanisms in real time but also establish a new generation of analytical tools capable of guiding personalized, adaptive immunotherapies.

## Outlook

Photonic biosensors are evolving from analytical tools into translational interfaces that bridge molecular readouts with therapeutic decision-making. By enabling real-time, label-free monitoring of immune cell interactions, cytokine secretion, receptor–ligand dynamics, and ncRNA signatures, these platforms can provide quantitative feedback on therapy specificity, efficacy, and patient-specific responses. Such capabilities are particularly relevant for advanced cancer immunotherapies, where dynamic cellular and molecular changes often determine therapeutic success or resistance.

The integration of photonic biosensors with microfluidic and lab-on-a-chip systems further enhances their potential, allowing high-throughput, single-cell, and longitudinal analyses under physiologically relevant conditions. This convergence creates opportunities to link molecular regulation, cell function, and treatment response directly, enabling the rapid optimization of molecular immunotherapies, CAR-T cell designs, and gene- or epigenetic-modulatory interventions. Moreover, multiplexed and high sensitivity biosensing can capture the complexity of immune and tumor signaling networks, providing a holistic view of therapeutic mechanisms that is difficult to achieve with conventional assays.

Looking forward, the combination of label-free optical biosensing with automated microfluidics, advanced biofunctionalization, and machine-learning-driven data analysis promises to transform the preclinical and clinical evaluation of cancer therapies. These integrated platforms could facilitate adaptive, feedback-informed immunotherapy strategies, guide personalized treatment regimens, and accelerate the translation of next-generation therapies from the bench to bedside. Ultimately, photonic biosensors are poised to become a cornerstone of precision oncology, providing the dynamic mechanistic insights required to realize the promise of immunotherapy in modern cancer care.
